# Sustained Release of Voriconazole Using 3D-Crosslinked Hydrogel Rings and Rods for Use in Corneal Drug Delivery

**DOI:** 10.3390/gels9120933

**Published:** 2023-11-28

**Authors:** Aiym Rakhmetova, Zhiqi Yi, Malake Sarmout, Leo H. Koole

**Affiliations:** National Engineering Research Center of Ophthalmology and Optometry, Eye Hospital, Wenzhou Medical University, Wenzhou 325027, China; aiym.rakhmetova@nu.edu.kz (A.R.); y18341468239@163.com (Z.Y.); malake.sarmout@wmu.edu.cn (M.S.)

**Keywords:** hydrogels, polymers, ocular drug delivery, fungal keratitis, cornea, voriconazole

## Abstract

Corneal disorders and diseases are prevalent in the field of clinical ophthalmology. Fungal keratitis, one of the major factors leading to visual impairment and blindness worldwide, presents significant challenges for traditional topical eye drop treatments. The objective of this study was to create biocompatible 3D-crosslinked hydrogels for drug delivery to the cornea, intending to enhance the bioavailability of ophthalmic drugs. Firstly, a series of flexible and porous hydrogels were synthesized (free-radical polymerization), characterized, and evaluated. The materials were prepared by the free-radical polymerization reaction of 1-vinyl-2-pyrrolidinone (also known as N-vinylpyrrolidone or NVP) and 1,6-hexanediol dimethacrylate (crosslinker) in the presence of polyethylene glycol 1000 (PEG-1000) as the porogen. After the physicochemical characterization of these materials, the chosen hydrogel demonstrated outstanding cytocompatibility in vitro. Subsequently, the selected porous hydrogels could be loaded with voriconazole, an antifungal medication. The procedure was adapted to realize a loading of 175 mg voriconazole per ring, which slightly exceeds the amount of voriconazole that is instilled into the eye via drop therapy (a single eye drop corresponds with approximately 100 mg voriconazole). The voriconazole-loaded rings exhibited a stable zero-order release pattern over the first two hours, which points to a significantly improved bioavailability of the drug. Ex vivo experiments using the established porcine eye model provided confirmation of a 10-fold increase in drug penetration into the cornea (after 2 h of application of the hydrogel ring, 35.8 ± 3.2% of the original dose is retrieved from the cornea, which compares with 3.9 ± 1% of the original dose in the case of eye drop therapy). These innovative hydrogel rods and rings show great potential for improving the bioavailability of ophthalmic drugs, which could potentially lead to reduced hospitalization durations and treatment expenses.

## 1. Introduction

The diagnosis and treatment of fungal keratitis can be problematic. The standard diagnostic technique requires scraping tissue from the edges and center of the corneal ulcer, followed by laboratory culture and microscopic analysis. This routine typically takes 1–2 weeks, during which therapy must be withheld [1]. Faster alternative methods, such as confocal microscopy in vivo and molecular techniques based on the polymerase chain reaction (PCR), have been developed but still await widespread acceptance and use [2,3].

Therapy of fungal keratitis is normally initiated by topical instillation of an antifungal drug such as natamycin, amphoterin B, or voriconazole [4,5]. However, surgical intervention is required in a considerable number of cases (around 40% [3]). Three factors hamper the success of early therapies based on eye drops: (i) the fact that a portion of an eye drop is spilled immediately after instillation due to blinking (spill rate can be >90%); (ii) clearance of the drug due to flow of the tear film toward the nasolacrimal duct; and (iii) the fact that the cornea poses a considerable barrier to diffusion of the drug toward the ulcer, especially when the ulcer has developed in deep-lying parts of the stroma [6,7,8]. The three factors suppress the so-called bioavailability of antifungal drugs from eye drops and, therefore, influence the efficacy of early treatment therapies for fungal keratitis. Hence, this is an important field of research. Much is to be expected from new innovative formulations that support sustained topical delivery of antimicrobial or antifungal agents close to corneal ulcers, as has been emphasized in numerous papers and reviews on the subject [9,10,11,12,13]. Such strategies almost inevitably revolve around biocompatible polymeric biomaterials, which should be engineered such that they will serve as a temporary reservoir for the drug, protect the drug against degradation as long as it resides within the depot, make sure that the drug is delivered locally in a predictable and sustained manner, and compensate (by sustained delivery) for efflux of the drug from the site of the lesion due to tear fluid flow across the surface of the cornea. In many approaches, such polymer biomaterials are hydrogels that can be either biodegradable or stable [14,15,16]. Biodegradable thermogel consisting of the triblock copolymer (PLGA-PEG-PLGA) or other gel formulations as an example, loaded with the voriconazole [17,18]. The formulation is injectable and transforms into a gel shortly after injection, i.e., once it reaches body temperature. Thereafter, the gel gradually decomposes to release the drug concomitantly [19,20]. Recently, the approach was evaluated in vivo (in horses), with promising results. Subconjunctival injection of the formulation space proved easy and safe, and the release of the drug during 48 h was observed [21].

Herein, we also utilize a novel synthetic structurally porous hydrogel biomaterial as a vehicle to achieve sustained drug release, aiming at the development of an alternative for eye drops during the early treatment of fungal keratitis. Our preconditions included that the technique must be practical, genuinely minimally invasive, and not leave any foreign material in the eye once delivery of the drug ends. Also, we wish to avoid covering the lesion, as would be the case if a drug-releasing contact lens system or a drug-releasing gel were used [22,23,24,25]. Based in part on our previous work with drug-loaded flexible rods, which were easy to insert and remove as they were placed in the conjunctival sac, we started developing flexible and biocompatible rod- or ring-shaped porous polymer constructs as depot/carriers. The key hypothesis is 2-fold: (i) the rods or rings can be placed temporarily around the site of infection, and (ii) porous cavities in the hydrogel biomaterial serve as encapsulation/depots for the sustained local release of an antifungal drug [26,27]. The pores contain the drug, either in crystalline form or formulated. We selected 3D-crosslinked poly(vinyl) pyrrolidone as our hydrogel. The previous studies of these materials not only encountered excellent biocompatibility features but also learned how to steer their physical-mechanical properties and swelling behavior [28,29,30]. Furthermore, we reasoned that poly(N-vinylpyrrolidone) (PVP) is a common material in ophthalmology; the polymer is a constituent of povidone/iodine formulations, which find widespread use in ophthalmology, even in the treatment of fungal keratitis [31]. Here, we describe the novel microporous variants of poly(N-vinylpyrrolidone). Interconnective micrometer-sized pores were created through the inclusion of low-Mw poly(ethylene glycol) (PEG) in the monomer mixture (as an inert filler) and wash-out of PEG post-polymerization. Here, we report preparation, characterization, and in vitro and ex-vivo studies with porous poly(N-vinylpyrrolidone) hydrogel rods and rings loaded with the antifungal agent voriconazole. To the best of our knowledge, this has not been reported so far. Slow drug release was observed in several experiments in vitro. Given their dimensions, strength/integrity, and flexibility, it is anticipated that the rods and rings can be placed without much difficulty on the corneal surface (close to, around, or on top of the site of infection/inflammation) and kept in place for several hours. This would support ongoing drug delivery from a depot near the site of infection/inflammation, during a time span that significantly exceeds the residence time of an eye drop, and in an essentially non-invasive manner. Removal of the rods or rings from the corneal surface should be easy as well. Much further development work is required for various reasons: (i) to make sure that voriconazole concentrations always remain within the therapeutic window (i.e., will never and nowhere reach toxic levels), and (ii) to attain control over the kinetics of the drug release, i.e., to optimize the therapeutic effect. Various handles can be pulled regarding (i), e.g., variation of porosity, size, and interconnectivity of the pores, crosslink density (which determines swelling aptitude of the polymer matrix), and payload of the drug.

## 2. Results and Discussion

### 2.1. Preparation of Hydrogel Rings and Rods

**Figure 1:**
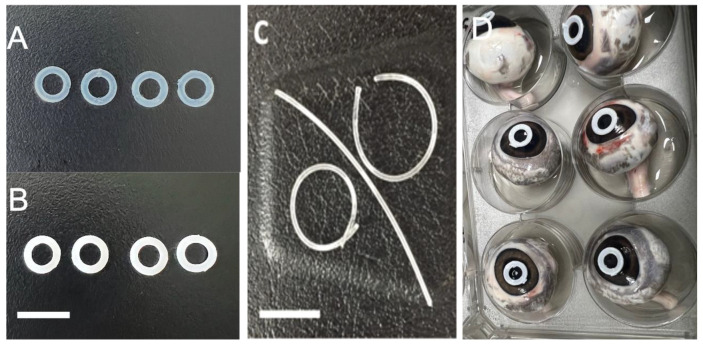
Photographs of (**A**) dry hydrogel rings, (**B**) wet hydrogel rings, and (**C**) representative rods. Scale bars correspond with 10 mm. Photograph (**D**) illustrates the ex-vivo experiment; six explanted porcine eyes are shown, each having a voriconazole-loaded hydrogel ring in touch with the surface of the cornea.

The chemical synthesis of the porous hydrogel biomaterials proceeded smoothly; the procedure is outlined in Section 4 (Materials and Methods); see below. After some exercise, the preparation of hydrogel rings and rods could be carried out without difficulties; Figure 1 shows the respective constructs. Note that the material was transparent and rubbery/soft immediately after polymerization, i.e., before washing and release of the PEG filler. After washing, the materials became white and opaque; when dried, the materials remained slightly opaque. Figure S2 shows the difference in the physical appearance of drug-loaded rods in wet and dry form.

It was shown that porous crosslinked hydrogels based on NVP can be made in defined shapes, such as rods and rings, which are easy to handle and feel soft and flexible. We believe this opens possibilities for adhering these rings or rods to the cornea, close to or even on top of the ulcer. One aspect worthy of consideration is the presence of a rod or ring at the corneal surface, which might introduce a sensation that differs from that of a soft contact lens and potentially affects comfort. These factors merit thorough in vivo investigation, and their outcomes must be carefully counterbalanced against therapeutic benefits.

### 2.2. Material Characterization

**Figure 2:**
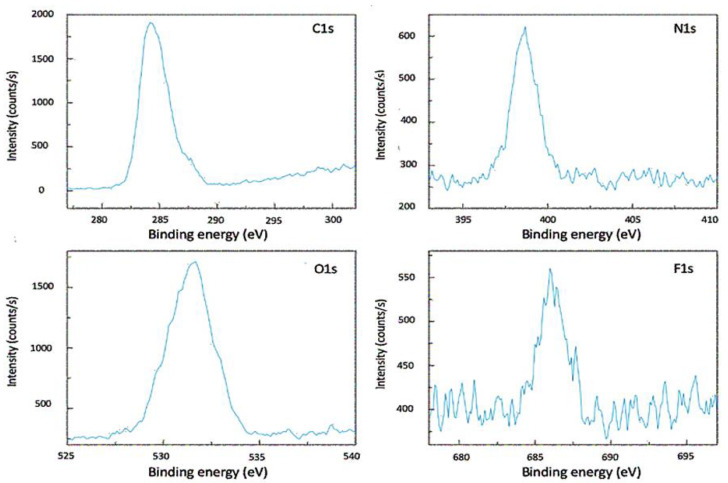
Sub-spectra from X-ray Photoelectron spectroscopy (XPS), as measured on the cross-section of a ring structure after loading with the drug voriconazole. The four panels show the C1s, N1s, O1s, and F1s peaks.

For the structural characterization of the hydrogels, we first turned to XPS (X-ray Photoelectron Spectroscopy). Figure 2 shows the characteristic sub-spectra of the drug-loaded hydrogel after drug loading and drying. The specimen was derived from one of our drug-loaded rods after cutting the sample at a 45-degree angle with its long axis. The cross-section sampled during XPS reveals information about the elementary composition of the inside of the rods. Note that the surface area of the elliptical cut is approximately 1.5 square mm, whereas the sample area for our XPS measurements was much smaller (0.04 square mm); the sample area was chosen close to the center of the cross-section. XPS peaks due to the presence of the elements C, N, and O were observed, which is in line with the composition of the polymer matrix (roughly: [C6H9NO]n).

The appearance of a fluorine peak in the XPS spectrum essentially proves qualitatively that the drug is present in the interior of the material. It was verified that the unloaded hydrogel material shows virtually identical C1s, N1s, and O1s peaks in the XPS spectrum without the appearance of a peak around 686 eV (F1s) (can be found in Figure S3).

**Figure 3:**
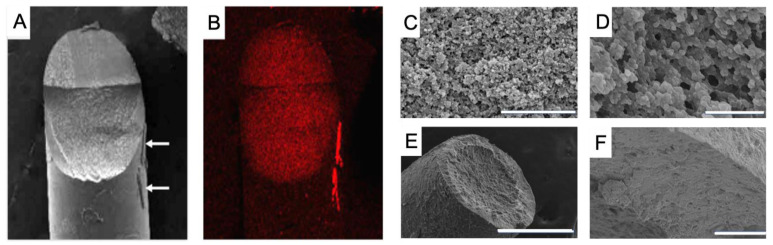
Images of the hydrogel: (**A**,**B**) Cross section SEM-EDS, the bar is 50.0 µm, (**C**,**D**) inner structure, the bar is 1.0–5.0 µm, (**E**,**F**) surface, the bar is 50.0–500 µm.

Independent corroboration of the presence of the drug in the interior pores of the drug-loaded materials was obtained with SEM EDX, a technique that allows scanning and mapping of surfaces in terms of elements present. Figure 3A,B shows the result of SEM EDX, revealing that voriconazole resides in the pores of the polymer network throughout the entire volume of the material. This observation indicates that swelling of the material during the 24-h incubation in the drug/DMSO solution “opened” the porous structure; the drug solution has been filling pores throughout the material’s entire volume.

After removal of the supernatant at the end of the soaking and immersion of the rod in excess water, rapid exchange of DMSO and water occurs, with concomitant crystallization of the drug in situ. Further studies with SEM revealed more insight into the structure and porosity of our hydrogel materials. Some typical examples of smooth surfaces in Figure 3E,F and open inner structures are shown in Figure 3C,D and Figure S4.

Mercury porosimetry was performed in order to characterize the materials further; the technique provides information not only on the porosity per se but also on the sizes and size distribution of the pores. Experimental data (Figure S5), measured on a hydrogel ring (prepared with 10% PEG, outer diameter 10 mm, inner diameter 6 mm, thickness 0.8 mm), revealed that the ratio Vv/Vt (i.e., void space: total space) is 0.30 with a pore-specific volume of 0.2478 cm^3^/g. A bimodal pore-size distribution was found: around half of the pore volume (54%) consists of pores having a diameter in the range of 5–25 nm, while the other approximate half (46%) of the void volume is pores in the diameter range of 2–240 μm.

### 2.3. Drug Release

**Figure 4:**
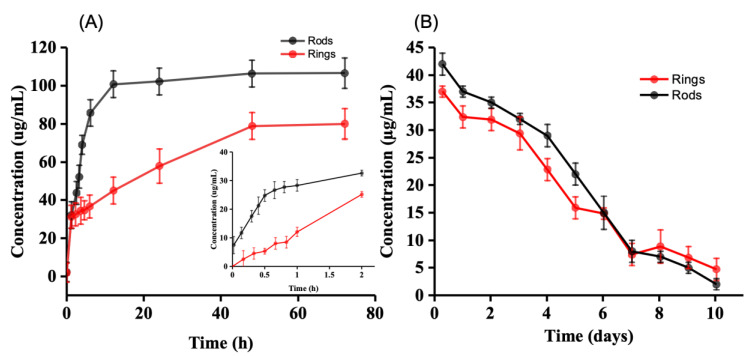
Cumulative release of voriconazole: (**A**) Concentration vs. time during first 3 days. Inside: during the first 2 h, (**B**) Release of voriconazole from the loaded rings with the refreshment of the supernatant.

The drug release kinetics from both rings and rods were analyzed by generating cumulative drug release profiles, aiming to comprehend the rate at which the drugs are released from the carrier. There was no significant difference between the release patterns of rings and rods. Figure 4A shows our data on the cumulative release of voriconazole from the drug-loaded hydrogel rings (outer diameter 10 mm, inner diameter 6 mm, thickness 0.8 mm, prepared with 10% porogen filler PEG). Release during the first 2 h (Figure 4 inside) is, to a good approximation, proportional with time. In other words, zero-order release kinetics are obeyed initially. Later, between 2 and 48 h, the release slows down. Later still, after 48 h, release practically stops (Figure 4B). For our intended application, we envisage that drug delivery during the first hours is most relevant, so it is important to focus especially on early release. It is of interest to calculate how much of the drug escapes from the ring during approximately the first 2 h.

In this stage, the release process obeys zero-order kinetics, i.e., the amount of drug set free is proportional to time. Release kinetics start to deviate from zero-order after 2 h, and a plateau is reached after, roughly, 2 days. The plateau can be explained, a priori, by (i) depletion of the drug reservoir or (ii) saturation of the supernatant. Figure 4 (inside) shows that the concentration reached 25 µg/mL for rods and 30 µg/mL for rings; with a sample volume of 9 mL, this means that 9 × 25 = 175 µg of the drug has been set free at this point (210 µg for rings, respectively). It is also of interest to compare this amount with the amount of voriconazole that is normally administered via an eye drop.

If we assume that the volume of an eye drop equals 50 µL and a voriconazole concentration of 1% (=10 mg/mL), then it follows that each eye drop introduces 155 µg of voriconazole into the eye. However, we know that a substantial amount of each eye drop is spilled almost immediately, primarily due to blinking [32].

If we assume the spilling rate to be 80%, then it follows that 30 µg of the drug is introduced into the tear film at the moment of instillation. This essentially summarizes the comparison we wish to make: the hydrogels releasing 175 µg of the drug linearly with time during 2 h versus a peak-type instillation of effectively 30 µg of the drug through an eye drop. 

Figure 4B shows data on voriconazole release from a slightly different experiment. Now, the ring and rods were left in the tube, while the supernatant (PBS) was exchanged regularly over a period of 10 days. Now, it is seen that drug release continues for a much longer time span (up to 10 days, approximately), with most of the drug being released during the first 4 days. The observation that the drug release ends relatively soon (i.e., after 2 days) in the first (cumulative release) experiment as compared to the second (medium-exchange) experiment may be explained by the poor solubility of voriconazole in PBS medium. Saturation may have been reached during the first experiment after 2 days; from this stage on, an equilibrium between encapsulated and dissolved drugs is established. Such a condition is not met when the medium is regularly refreshed; release will then continue, albeit slower and slower until the reservoir is depleted. Based on the data, it was calculated that approximately 210 µg of the drug had been released in total. This compares favorably with the 175 µg release during the first experiment (vide supra).

**Figure 5:**
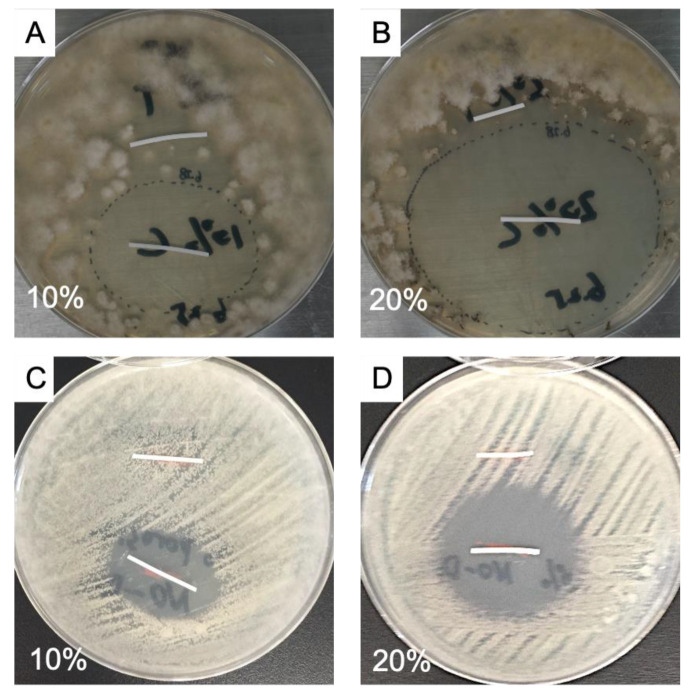
Photographs of cultured fungi on an agar layer in a Petri dish and hydrogel rods: (**A**,**B**) *Aspergillus* spp., (**C**,**D**) *Fusarium* spp. The lower rods are drug-loaded, and above are unloaded controls.

Figure 5 shows images from a different kind of controlled release experiment, which is qualitative, albeit, in our opinion, illustrative. Two so-called clinical isolates, i.e., fungi, which were isolated from patients with fungal keratitis, were cultured on an agar layer in a Petri dish. One was *Aspergillus* spp., and one was *Fusarium* spp. The fungi were evenly smeared out over the dish’s surface and allowed to grow under standard conditions in an incubator for 24 h. Then, a drug-loaded and a drug-free rod were placed on the dish; the rods were placed in parallel at a distance of approximately 3 cm. Figure 5A shows a dish on which *Aspergillus* spp. was cultured. The image was taken 1 day after the placement of the two rods. The lower rod is loaded with voriconazole, whereas the upper one is the drug-free control. Around the lower rod, an elliptic zone has formed in which the fungus is killed. No such “zone of inhibition” is observed around the upper rod. Figure 5B shows the analogous situation, but now the drug-containing rod has a porosity of 20%, i.e., the drug loading is higher. The elliptic zone around the rod has expanded considerably due to increased release and farther outward diffusion of the drug, thus killing a larger part of the fungus. Figure 5C,D are analogous, but now for *Fusarium* spp. In Figure 5C, we again clearly see an elliptic zone of inhibition around the drug-loaded (lower) rod and no inhibition at all around the control. Figure 5D displays a larger elliptic zone of inhibition due to the use of a 20% porous hydrogel and its higher drug-loading capacity. 

### 2.4. Ex-Vivo Assessment with Porcine Eyes

The bioavailability of the voriconazole drug was assessed by measuring its concentration in the corneas of porcine eyes (*n* = 8) after a 2-hour period (see Figure 1D). It is noteworthy that the penetration of voriconazole through porcine corneas is presumed to be somewhat reduced compared to human corneal penetration due to the greater thickness of the porcine corneal epithelial layer [33]. The study’s results, as shown in Table 1, reveal that, after 2 h, the average amount of voriconazole detected in the corneas was 60.31 ± 6.0 and 42.93 ± 4.8, suggesting a drug penetration efficiency of 35.8 ± 3.2% and 25.1 ± 2.5%, which was calculated according to Equation (1).

(1) $$\frac{Drug\;amount\;found\;in\;the\;cornea\;(\mu g)}{Drug\;amount\;loaded\;(\mu g)}\times 100\%.$$

When comparing the loaded rings to an equivalent concentration eye drop, following literature guidelines [34,35], it’s observed that one drop of 50 µL (containing 1% voriconazole) delivers an average of 6 ± 1.5 µg after 2 h. This accounts for approximately 3.9% of the administered dose. Therefore, it becomes evident that voriconazole-loaded hydrogel inserts deliver a significantly higher amount of voriconazole compared to equivalent concentration eye drops at the same time point.

The results from Table 1 demonstrate that, overall, these sustained-release hydrogel rings exhibit enhanced drug bioavailability, leading to a 10-fold increase in the amount of voriconazole detected in the cornea after two hours of dosing. Achieving high concentrations in the target tissue so rapidly (within 2 h) following the insertion of voriconazole-loaded rings may obviate the need for concurrent topical voriconazole treatment, especially in severe cases of fungal keratitis. Additionally, the hydrogel device enables a continuous supply of the drug to the eye from the applied site, ensuring delivery until all the drug contained in the hydrogel has been exhausted, even beyond the 2-h timeframe used in the experiment. The sustained drug infusion could lead to increased effectiveness while requiring a smaller dosage when compared to conventional treatment using topical voriconazole. Thus, hydrogel inserts hold promise not only for prolonged drug release but also for expediting the treatment of fungal infections.

### 2.5. Cytocompatibility of Hydrogels

**Figure 6:**
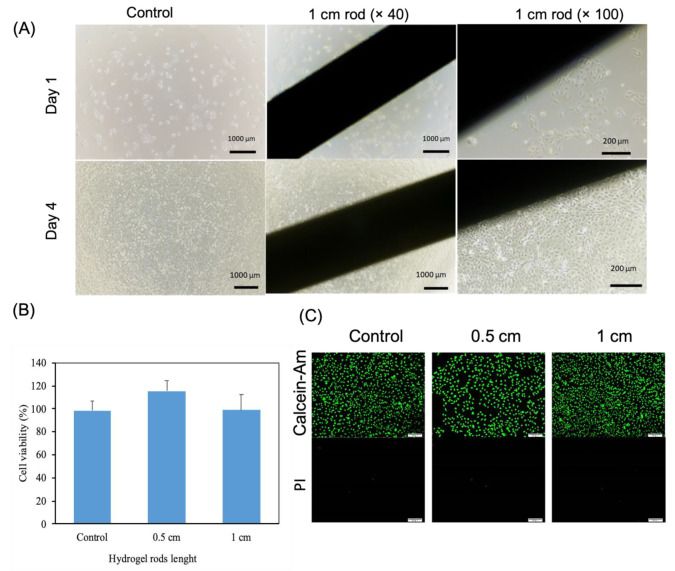
Cell viability of hydrogel rods and control group: (**A**) Photomicrograph images of culture well plate after 24 h and 96 h of cell seeding, (**B**) CCK-8 analysis, (**C**) Cell vitality staining after 24 h. The scale bar represents 100 µm.

To assess the compatibility of rings and rods with cellular growth, we utilized HCEC cells as a model for conducting a live/dead experiment and cell proliferation analysis. As shown in Figure 6C, substantial numbers of living cells (living cells emit green fluorescence when excited with light at a wavelength of 490 ± 10 nm, whereas dead cells exhibit a red fluorescence when excited at a wavelength of 545 nm) were observed in rods measuring both 0.5 and 1 cm in length after 24 h of culture, with barely any detectable dead cells. These findings unequivocally validate that hydrogels do not exert any negative effects on cell viability and proliferation, underscoring their remarkably low cytotoxicity. To further quantify cell proliferation when exposed to the hydrogels, we employed the CCK8 assay for cytocompatibility assessment. As illustrated in Figure 6B, there were no significant fluctuations in optical density (OD) values across all groups when compared to the control group, providing additional evidence of the outstanding cytocompatibility of the material. The hydrogel did not induce any considerable cytotoxicity even after 4-day periods of direct contact, and cell proliferation is highly comparable with the control plate (Figure 6A and Figure S6). This is an essential requirement to make the step to subsequent in vivo investigations, first with animal models and later with patients.

## 3. Conclusions

The treatment of corneal ulcers in ophthalmology remains a critical challenge, necessitating innovative concepts and techniques to improve therapeutic outcomes. One of the primary obstacles is the need to enhance the local bioavailability of antifungal agents through sustained and controlled drug delivery to the affected area. The findings presented in this study offer promising insights for potential translation into clinical applications. The drug-releasing rods and rings, developed as part of this research, exhibit considerable potential by accommodating adequate quantities of voriconazole. Notably, the in vitro release of voriconazole from the rings demonstrates a dosage range comparable to that of traditional eye drops, ranging from 175 to 210 µg. Furthermore, the charged hydrogels exhibited continuous and time-proportional drug release over 2 h. This stands in stark contrast to conventional eye drops, which are associated with a “peak supply” of the drug, followed by a rapid decline due to tear film flow. Ex vivo analyses conducted on porcine eye corneas further reinforce the superiority of the porous hydrogel-based approach. Particularly important is the observation that, after 2 h of application, the hydrogel ring retrieves a significant portion of the original dose (35.8 ± 3.2%) from the cornea, in contrast to the minimal retrieval (3.9 ± 1%) associated with conventional eye drop therapy. This enhanced and prolonged local drug release aligns well with pharmacological objectives, holding the potential for improved treatment outcomes. It is worth noting that while the concept of extended drug release holds great promise, practical applications require further investigation. The utilization of contact lenses loaded through the transfer technique offers convenience, but its effectiveness may differ, as preliminary data suggest. Ongoing research within our laboratories and clinical settings is dedicated to resolving these issues.

## 4. Materials and Methods

### 4.1. Synthesis of Materials

Two different porous hydrogels were prepared, one with 10% PEG porogen filler and one with 20% PEG porogen filler. Preparation of the 10% PEG hydrogel started with NVP (3.45 g, 0.03 mol), crosslinker (1.5 g, 4.5 mmol), initiator (1.05 g, 5 mmol), and PEG1000 (0.6 g), which were first transferred into a 50-mL round bottom flask and then carefully mixed through magnetic stirring. Approximately 20 min were required to achieve complete dissolution of the PEG. This formulation, in which the ratio mass (NVP + crosslinker + initiator): mass filler = 10:1, was coined as 10% PEG hydrogel. Preparation of the 20%-PEG hydrogel was analogous, except that the double amount of PEG1000 was added (1.2 g), i.e., the ratio mass (NVP + crosslinker + initiator): mass filler was 10:2. For the preparation of the porous hydrogel rods, we transferred the monomer mixture (60 mL) into straight glass capillaries (approximately 10 pieces, length 80 mm, inner diameter 1.0 mm), which were closed on one end. Retrograde filling of the capillaries, using a drawn glass pipette, was applied to avoid the inclusion of air bubbles. The synthesis procedure employed a water bath as the main heating method. First, the capillaries filled with the desired solution were carefully immersed in a water bath set to a specific temperature of 80 °C. The capillaries were then left undisturbed within the water bath for a precisely timed duration of 60 min, allowing the desired reactions to occur. Then the capillaries were removed from the oven and allowed to cool to room temperature overnight. The next morning, using protective gloves, the capillaries were carefully broken. After some exercise, this released the transparent hydrogel rod into intact structures that were practically free from glass. The rods were immersed in water, which detached the last glass pieces and allowed PEG and unreacted NVP to dissolve. The hydrogel materials imbibed water, as was clear from the occurrence of a color change (from transparent to white-opaque) and slight swelling.

The rings were prepared with specially designed Teflon (Polytetrafluoroethylene, PTFE) molds, made via lathe-milling in our workshop (Figure S1). Four different molds were used, each one derived from a PTFE block with dimensions of 76 × 25 × 6 mm. The corresponding characteristics of these molds are summarized in Table 2 below, which includes mold number (#) and specific parameters. 

The molds were carefully filled with a monomer mixture, transferred into a preheated oven (80 °C), kept inside for 60 min, and then allowed to cool. Materials were removed from the molds through careful manipulation/lifting of the rectangular parts (thin forceps), i.e., without touching the circular parts. Loosened constructs were immersed in excess water and allowed to imbibe water, swell, and release PEG and unreacted monomers. This transformed the constructs into soft, rubbery materials from which the rectangular parts could be cut off easily with a razor. Rods and rings thus obtained were dried in two steps: (i), on a Petri dish in ambient conditions (2 days), and (ii), in a desiccator under moderate vacuum.

### 4.2. Material Characterization

The morphology and microstructure were examined using scanning electron microscopy (SEM, Hitachi SU8010-5000, Tokyo, Japan). Before their analysis, samples were sputter-coated with platinum (Leica EM ACE600, Wetzlar, Germany). X-ray photoelectron spectroscopy (XPS) measurements were performed externally by the company HuaYou Testing and Technology Service Ltd., Guangzhou, China. Quantitative analysis of the porous structure was done with mercury intrusion porosimetry, using a POREMASTER 60 instrument (Quantachrome Instruments, Boynton Beach, FL, USA).

### 4.3. Drug Release In Vitro

Spectrophotometric analysis of voriconazole was done according to the literature [9,36]. We verified that the absorption maximum is at 256 nm (PBS, pH 7.4) and that there is proportionality (Lambert-Beer law) between extinction and voriconazole concentration up to 80 mg/mL; a linear calibration line was constructed. Several experiments were done to study the release of voriconazole from our porous hydrogel rods and rings in vitro. The first set of experiments was done with 95 identical hydrogel rings (dimensions: outer diameter = 10 mm, inner diameter = 6 mm, height = 0.8 mm; prepared with 10% PEG), which were drug-loaded as described above. All of these rings were incubated with 1.0 mL of PBS in a 7-mL glass tube. Tubes were immersed in a water bath (37 °C). Supernatants were pipetted out quantitatively in 5-fold at the following 19-time points: 10′, 20′, 30′, 40′, 50′, 1 h, 2 h, 3 h, 4 h, 5 h, 6 h, 7 h, 8 h, 12 h, 24 h, 48 h, 72 h, 96 h, and 120 h. Each supernatant was mixed with 9 mL of fresh PBS, homogenized, and stored at 4 °C until spectrophotometric analysis. 

The second set of experiments used six of the same drug-loaded rings. They were incubated with 10 mL of PBS and kept at 37 °C for 10 days. Now, the supernatants were removed and replaced with fresh PBS at the time points: 4 h, 8 h, 16 h, 24 h, 2 d, 3 d, 4 d, 5 d, 6 d, 7 d, 8 d, 9 d, and 10 d. Supernatants were stored at 4 °C until spectrophotometric analysis.

In vitro experiments focused on the antifungal activity of released voriconazole were performed with drug-loaded rods; specimens with 10% porosity and 20% porosity were used. In these experiments, clinical isolates of *Fusarium* spp. or *Aspergillus* spp. were used, i.e., these fungi had been isolated from patients’ infected eyes, and preserved for research purposes. The densities of the conidial or sporangiospore suspensions were read on a spectrophotometer (530 nm, 1.0 cm light path) and adjusted to an optical density in the range of 0.09–0.13 for *Aspergillus* spp. and 0.15–0.17 for *Fusarium* spp. [37]. Fungi were first spread on the agar surface (inoculating loop, streak plate method). It was attempted to distribute the fungi as homogeneously as possible over the surface. Next, both a drug-loaded and an unloaded rod (control) were placed on the agar surface; the rods were placed in parallel, at approximately 30 mm distance from each other, equidistant from the dish’s center. Then, the dishes were placed in an incubator (35 ± 0.5 °C, 60% relative humidity) and examined/photographed daily. Zones of inhibition around the drug-loaded rods could be observed clearly.

### 4.4. Ex-Vivo Assessment with Porcine Eyes

Porcine eyes were obtained from a local abattoir on the day of slaughter. The eyes were transported at 4 °C and used for experimentation within 6 h of collection. Prior to the experiment, a thorough inspection was conducted to check for any damage or scarring. Excess fat was carefully removed using scissors. Subsequently, each eye was placed in a separate well of a 6-well plate, with the cornea facing upward. To maintain hydration, 1 mL of phosphate-buffered saline (PBS) was added to each well. The plate was then immersed in a water bath set at 35 ± 1 °C for 10 min. Afterward, drug-loaded rings were applied to each eye, as shown in Figure 1D. In the study, both dry and pre-soaked hydrogel rings were employed (*n* = 8). To simulate a steady tear flow, freshly prepared simulated tear fluid (STF) was instilled into each eye at a rate of 2.4 mL/hour. This was achieved by slowly dripping 200 μL of STF every 5 min. The eyes were maintained in this condition for a total of 2 h [9,38]. Following that, the eyes were removed from the water bath, and the drug-loaded rings were carefully removed. Subsequently, each cornea was dissected using surgical scissors and forceps. Each cornea was placed in a 5 mL glass vial containing 2.00 mL of high-performance liquid chromatography (HPLC)-grade methanol. The vials were placed on a shaking incubator (Bluepard, Shanghai, China) operating at 150 rpm and maintained at room temperature overnight. On the following day, each cornea was removed from the vial, and the methanol solution was transferred to auto-sampler vials in preparation for HPLC analysis.

### 4.5. HPLC Analysis

HPLC was performed using an Agilent 1260 Infinity II HPLC system by Agilent Technologies (Waldbronn, Germany), equipped with ChemStation software (LTS 01.11). The HPLC system consisted of a G7111A 1260 Quaternary Pump VL, a G7114A 1260 Variable Wavelength Detector (VWD), and a G7129A 1260 vial sampler. The chromatographic column used was a Poroshell 120 EC-C18 (4.6 mm I.D. × 150 mm, 4 μm) by Agilent Technologies (Waldbronn, Germany).

For the detection of voriconazole, a mobile phase was prepared, comprising an ammonium acetate buffer (pH 4.4), acetonitrile, and methanol in a ratio of 55:30:15. The UV detector was set at a wavelength of 250 nm, with each injection having a volume of 10 µL. The flow rate was maintained at 1 mL/min, and the total run time was 9 min. To establish the calibration curve, standard solutions of voriconazole were employed, spanning a concentration range of 5–150 µg/mL with a mean R^2^ value of 0.9999. Notably, the retention time for both the calibration curve and the drug release profile was approximately 7.3 min.

### 4.6. Cytocompatibility of Hydrogels

Assessing the biocompatibility of biomaterials is an essential criterion that determines their applicability for various biomedical uses [39]. The biocompatibility of the selected hydrogel was studied with several in vitro assays, including direct contact of the biomaterial with cultured human cornea epithelial cells, cell count analysis, and cell viability assays. 

#### 4.6.1. Cell Count Kit 8 (CCK-8)

To evaluate the biocompatibility of the hydrogel material, in vitro cytotoxicity assays were conducted using the CCK-8 assay [40]. The study aimed to investigate the impact of the hydrogel of two different lengths (0.5 cm and 1 cm) on the viability of human corneal epithelial cells (HCEC). The experiment consisted of three groups, namely, the control group, the experimental group with 0.5 cm of hydrogel, and the experimental group with 1 cm of hydrogel. Cells were seeded in 96-well plates with approximately 5000 cells per well and incubated at 37 °C with 5% CO_2_ for 24 h to allow for cell adhesion. Subsequently, the old medium was removed, and the experimental groups were treated with 100 μL of culture medium containing hydrogel for 24 h, while the control group was treated with a clean culture medium. The plates were then incubated for an additional 24 h under the same conditions. After incubation, the original culture medium was aspirated, and 100 μL of culture medium with CCK-8 solution was added to each well at a ratio of 10:1 (culture medium: CCK-8 solution). The plates were further incubated for 1.5 to 2 h. The optical density (OD) value was measured at 450 nm using a microplate reader.

#### 4.6.2. Live-Dead Assay

Firstly, the dye solution was prepared by mixing Calcine AM, PI, and DPBS in a ratio of 2:3:1000. The solution was then dosed at 500 μL per well. Next, cells were inoculated into a 24-well plate at a density of 3 × 10^4^ cells/well with 1 mL medium/well. The plate was placed in a cell incubator (37 °C, 5% CO_2_) for 24 h. 

After 24 h, the culture medium was replaced with a hydrogel-soaked medium, and the plate was further incubated for 24 h. The live/dead cell staining was performed by aspirating the culture medium from each well, mixing it with dead cells, and transferring it to a 1.5 mL EP tube. The tube was then centrifuged at 1000 rpm for 4 min, and the supernatant was aspirated to leave dead cells at the bottom. The plate was washed twice with DPBS solution, and the DPBS from each well was collected in a 15-mL EP tube. The DPBS was mixed with dead cells left at the bottom after centrifugation. The prepared staining solution was added to the EP tube filled with dead cells, mixed thoroughly, and then 500 μL of the staining solution mixed with dead cells was added to each well. The plate was incubated in a cell incubator (37 °C, 5% CO_2_) for 15 min.

Finally, cell viability staining images were captured using a fluorescence microscope. Living cells appeared green under excitation at a wavelength of 490 ± 10 nm, while dead cells appeared red under excitation at a wavelength of 545 nm. The results were analyzed to determine the survival rate of cells in the presence of hydrogel.

#### 4.6.3. Cell Proliferation in Direct Contact

The procedure was carried out to evaluate the effects of 1 cm hydrogel in direct contact with the cell culture. Photomicrographs were conducted to examine the morphological changes of the cells under the microscope. This method provides a visual representation of cellular morphology and can reveal any alterations induced by the addition of the hydrogel. Such analysis can provide valuable insights into the influence of hydrogel on cell growth and aid in determining the optimal conditions for cell culture.

Following the preparation of a 1 cm hydrogel and the calculation of cells, a 24-well plate was inoculated with the calculated cell suspension at a density of 3 × 10^4^ cells/well with 1 mL of medium/well. Positive controls were used in the experiment by utilizing wells containing fresh medium in the absence of any biomaterials. The plate was incubated in a cell incubator (37 °C, 5% CO_2_) for 24 h. After 24 h, the old medium was removed and replaced with a new medium soaked in 1 cm hydrogel. The cells were then cultured for an additional 24 h. Afterward, they were magnified 40 times under a microscope for observation and photomicrograph analysis.

## Figures and Tables

**Table 1:** Comparison of voriconazole amount that has been absorbed by porcine corneas after hydrogel rods and eye drops.

Hydrogel Rings (after 2 h), µg		
Sample	Dry form	Wet form
1	40.232	55.92
2	36.296	57.43
3	44.716	66.406
4	43.748	56.034
5	36.512	52.828
6	48.324	64.72
7	45.084	59.43
8	48.522	69.734
Mean ± SD	42.93 ± 4.8	60.31 ± 6.0
Bioavailability	25.1 ± 2.5%	35.8 ± 3.2%
1% Voriconazole eye drops (after 2 h), µg [34]		
	Drug amount	Bioavailability
Mean ± SD	6 ± 1.5	3.9 ± 1.0%

**Table 2:** Dimensions of the molds, obtained by computer-controlled lathe/milling of PTFE blocks.

Mold #	Outer Diameter, mm	Inner Diameter, mm	Depth, mm
1	10.0	6.0	1.0
2	10.0	6.0	0.8
3	9.0	5.0	1.0
4	9.0	5.0	0.8

## Data Availability

Data is contained within the article and Supplementary Materials.

## Supplementary Materials

The following supporting information can be downloaded at: https://www.mdpi.com/article/10.3390/gels9120933/s1, Figure S1: Schematic representation and real photo of Teflon mold; Figure S2: Photo of wet and dry form of rod; Figure S3: XPS spectra of drug-free hydrogel rings; Figure S4: SEM images of drug-free hydrogel rings; Figure S5: Mercury porosimetry analysis chart of hydrogel rings; Figure S6: Photomicrograph images of the culture well plate (control) and the 1 cm rods after 48 and 72 h of cell seeding.

## List of Abbreviations

CCK-8	Cell Counting Kit-8
DMSO	Dimethyl Sulfoxide
HCEC	Human Corneal Epithelial Cells
HPLC	High-Performance Liquid Chromatography
NVP	No Visible Particles
PBS	Phosphate-Buffered Saline
PEG1000	Polyethylene Glycol 1000
PLGA	Polylactic-co-glycolic acid
PTFE	Polytetrafluoroethylene
PCR	Polymerase Chain Reaction
SEM	Scanning Electron Microscope
SEM-EDX	Scanning Electron Microscope with Energy Dispersive X-ray Spectroscopy
UV-Vis	Ultraviolet-Visible Spectroscopy
XPS	X-ray Photoelectron Spectroscopy
