# Supplementary material for: Sustained Release of Voriconazole Using 3D-Crosslinked Hydrogel Rings and Rods for Use in Corneal Drug Delivery
Source: Gels. 2023 Nov 28;9(12):933. doi: 10.3390/gels9120933 (PMC10742393; doi:10.3390/gels9120933)
Supplement: Supplementary file 1 [file gels-09-00933-s001.zip › gels-2676106-supplementary.pdf]

Supplementary Materials

Sustained Release of Voriconazole Using 3D-Crosslinked Hydrogel Rings and Rods for Use in Corneal Drug Delivery

Aiym Rakhmetova, Zhiqi Yi, Malake Sarmout and Leo H. Koole *

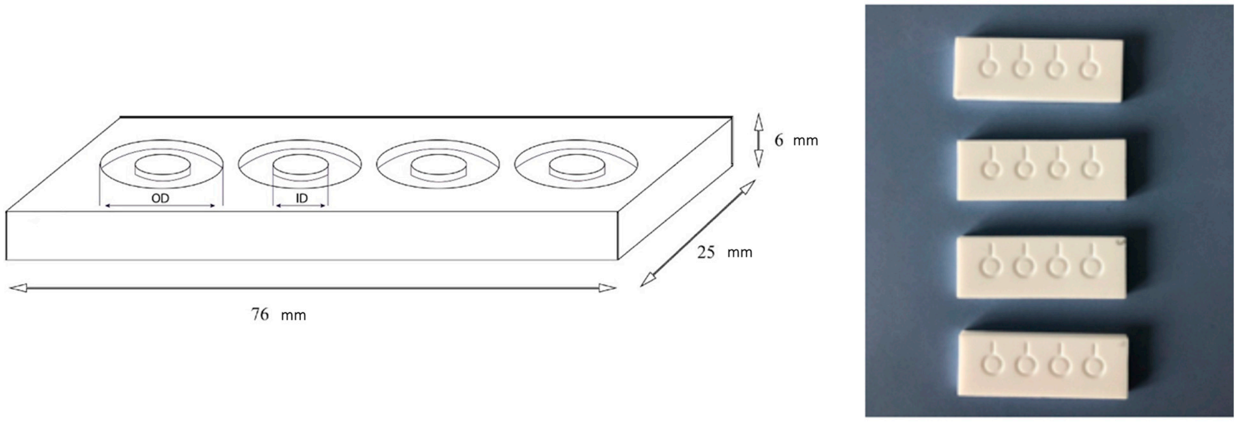

(**a**) (**b**)

Figure S1. a) Schematic representation b) real photo of Teflon mold.

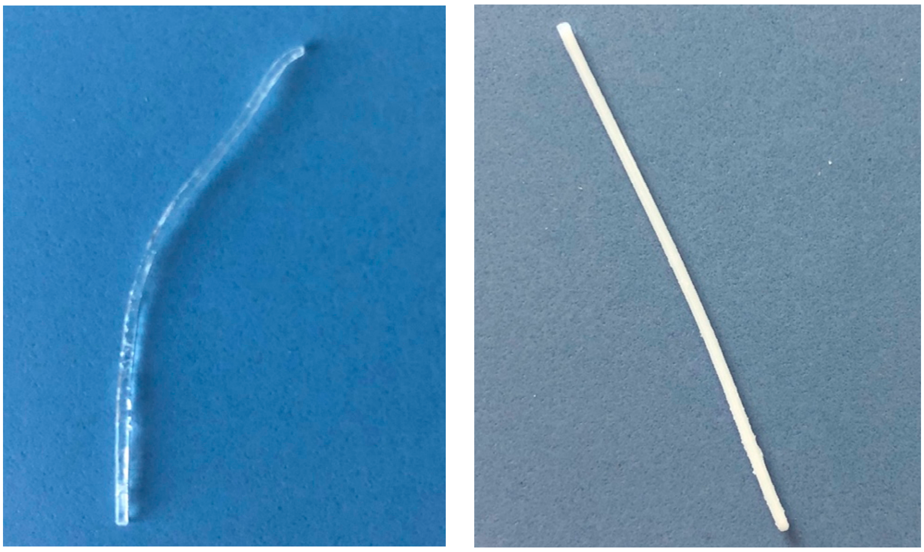

(**a**) (**b**)

Figure S2. Synthesized drug-loaded polymer rods shown in different states: a) Dry form; b) Wet form.

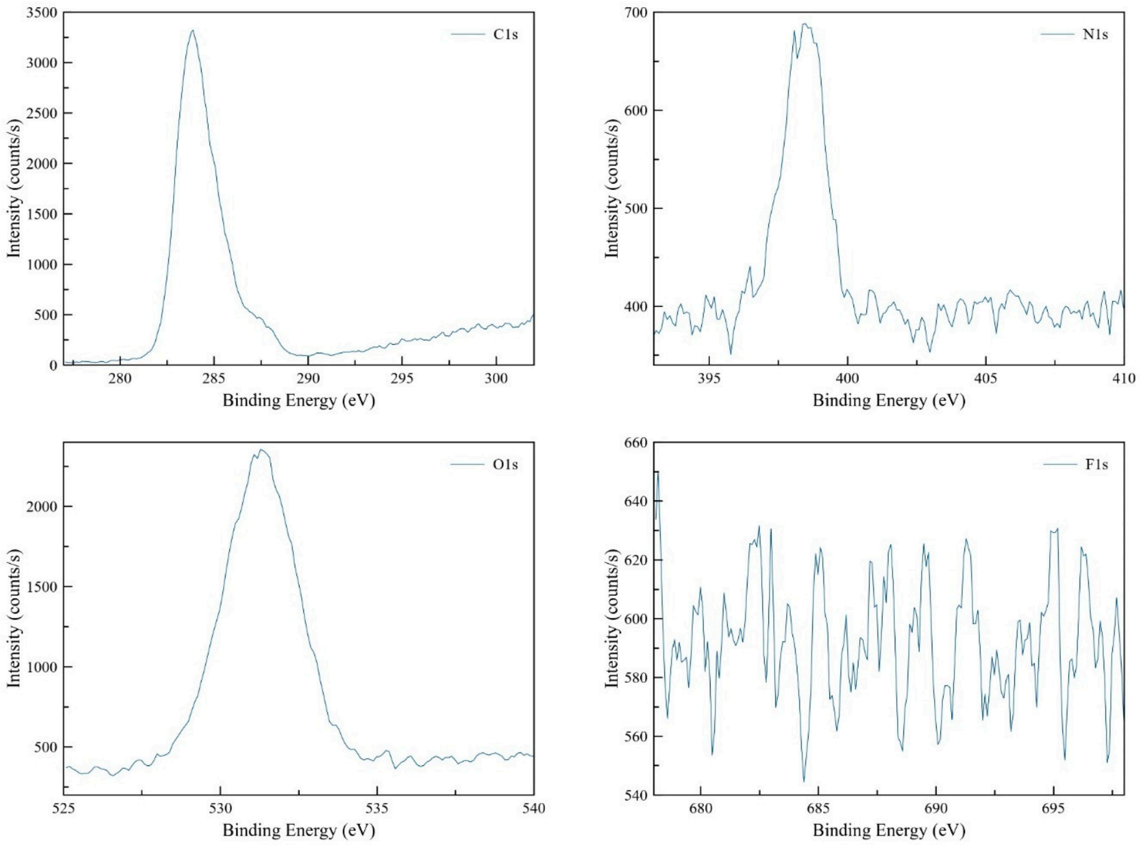

Figure S3. XPS spectra of drug-free hydrogel rings.

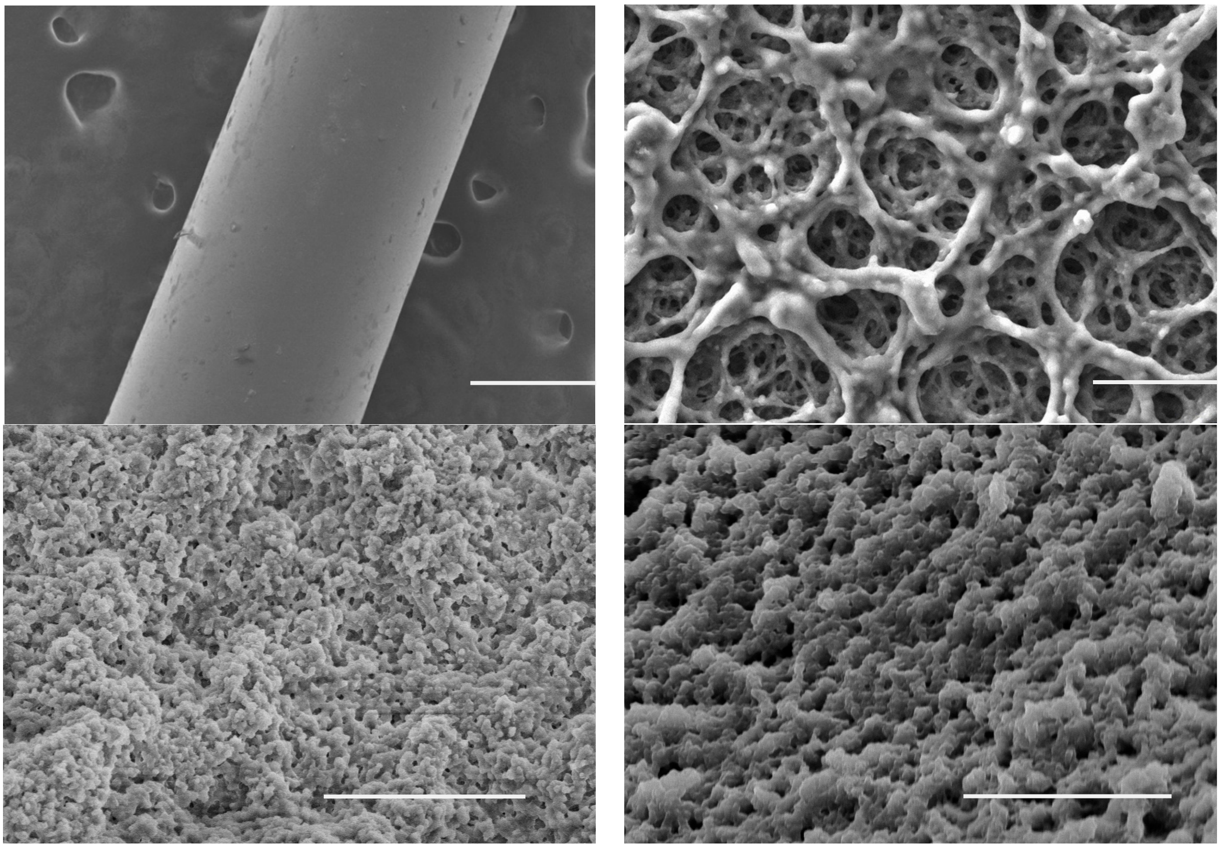

Figure S4. SEM images of drug-free hydrogel ring (Scale bar in each panel represents 5 μm).

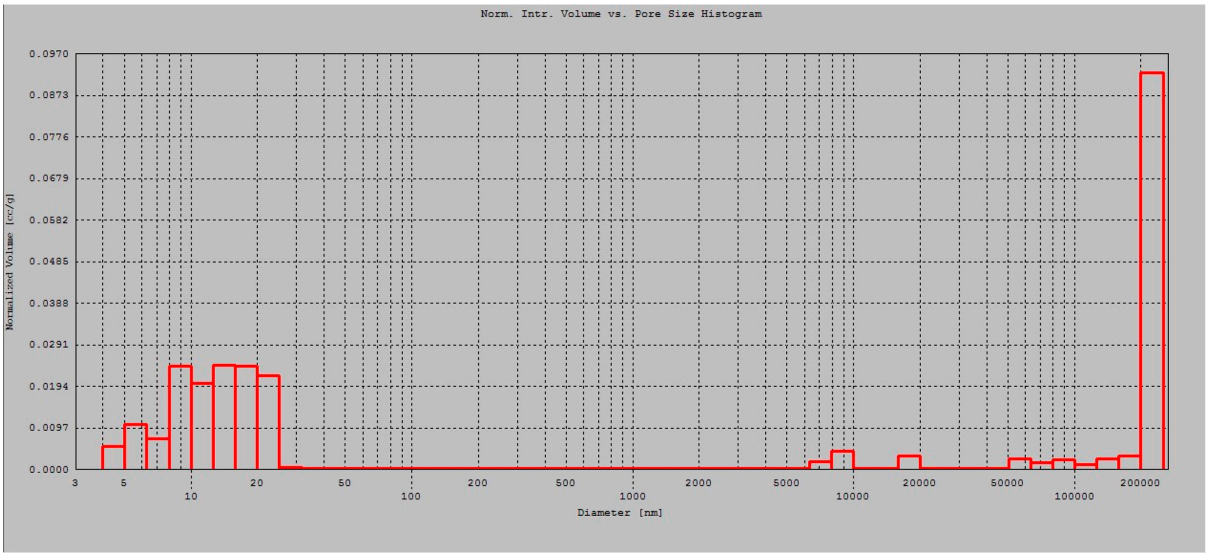

Figure S5. Mercury porosimetry analysis chart of hydrogel rings.

After 48 h

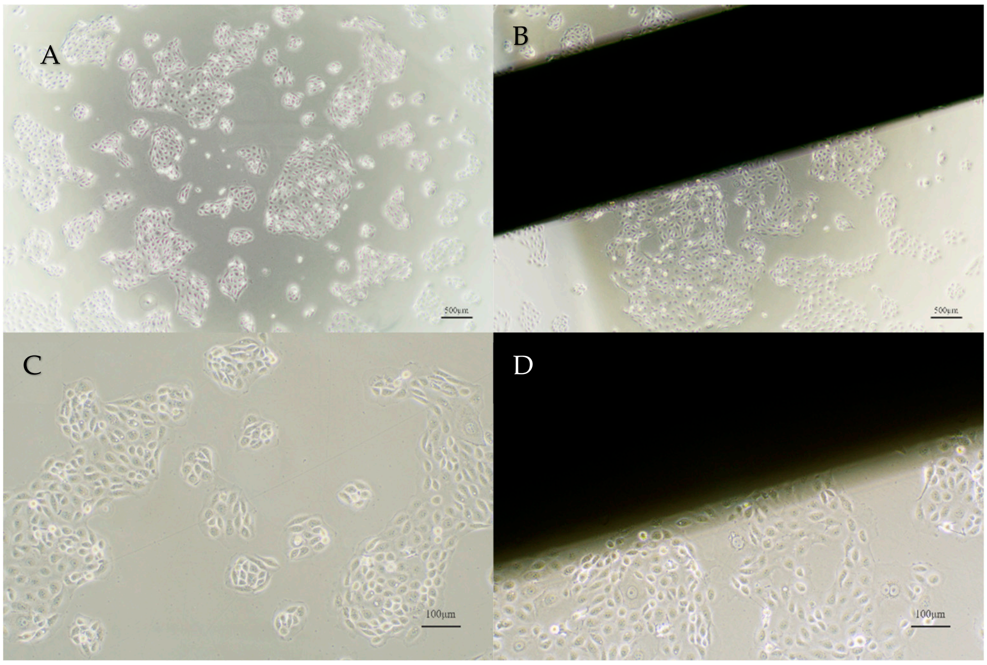

After 72 h

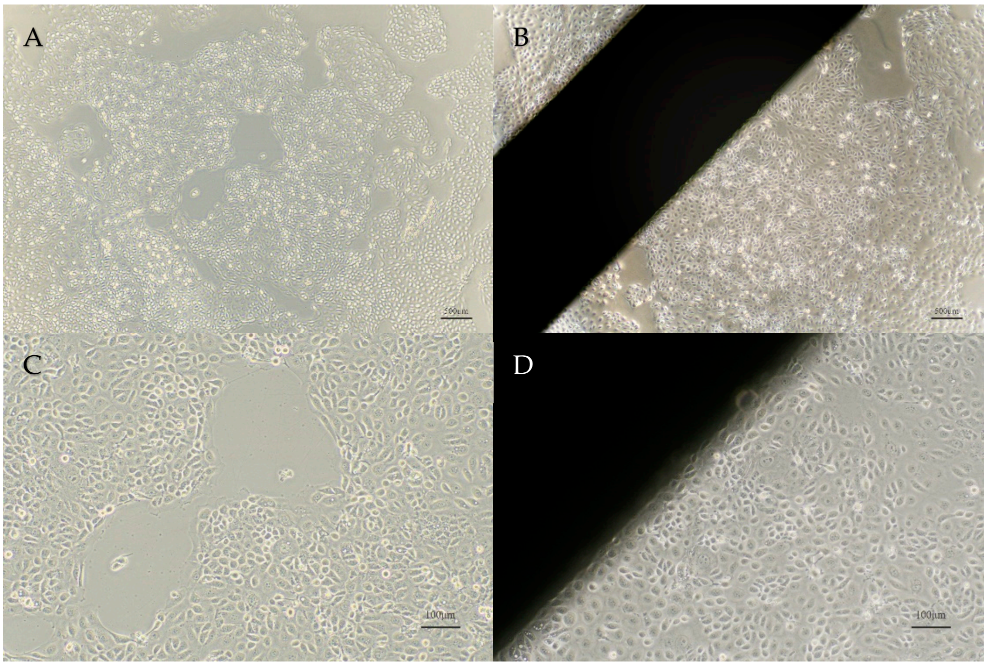

Figure S6. Photomicrograph images of culture well plate (control) and the 1 cm rods after 48 h (above) – 72 h (below) of cell seeding. A-B**:** Images taken with 10× magnification**,** C-D**:** Images taken with 40× magnification.
